# Scientific challenges for precision public health

**DOI:** 10.1136/jech-2019-213311

**Published:** 2020-01-23

**Authors:** Frank Kee, David Taylor-Robinson

**Affiliations:** 1 Centre for Statistical Science and Operational Research (CenSSOR), Queen's University Belfast, Belfast, UK; 2 Public Health and Policy, University of Liverpool, Liverpool, UK

**Keywords:** public health policy, epidemiology, randomised trials

## Abstract

The notion of ‘precision’ public health has been the subject of much debate, with recent articles coming to its defence following the publication of several papers questioning its value.

Critics of precision public health raise the following problems and questionable assumptions: the inherent limits of prediction for individuals; the limits of approaches to prevention that rely on individual agency, in particular the potential for these approaches to widen inequalities; the undue emphasis on the supposed new information contained in individuals’ molecules and their ‘big data’ at the expense of their own preferences for a particular intervention strategy and the diversion of resources and attention from the social determinants of health.

In order to refocus some of these criticisms of precision public health as scientific questions, this article outlines some of the challenges when defining risk for individuals; the limitations of current theory and study design for precision public health; and the potential for unintended harms.

‘Precision’ public health (PPH)^1^ is conceptualised as a means of improving population health through the use of new technologies, particularly genomics and digital, which would guide public health practice by generating more individually tailored interventions and policies. The concept of PPH has been the subject of much recent debate, with the Editor of the Lancet recently coming to its defence following the publication of several papers questioning its value.^2 3^ In this essay, we frame some key challenges for PPH as unanswered scientific questions, in an attempt to move the discussion forward.

While numerous articles use the term PPH,^4^ there is a lack of a universally agreed definition,^5^ with Khoury recently suggesting PPH as a cover-all term for ‘the next generation public health’

Given the vagueness attending this definition, it is important that we declare, at the outset, the focus of this article. We do not focus on PPH applications, which we believe to be scientifically uncontentious, for example, using information on the genetic profiles of microbes and exposed individuals in communicable disease outbreak detection and management.^6^ Nor do we address the curation and analysis of ever more detailed data on populations at finer geographical scales for the purpose of targeting public health interventions on communities,^7^ which is, in our view, little more than conventional public health, but with richer data.

Instead, we take our cue from the PPH emphasis in several policy documents in the UK, notably in the recent Green paper on prevention,^8^ which spotlights targeted or personalised interventions for lifestyle and behavioural change following individual genetic or digital profiling, for example, using smartphone apps. This version of PPH is our main focus. While the Lancet editor warns against ‘uncritical techno-optimism’ with regard to PPH, he also decries unsustainable ‘pilotitis’, an excess of pilot studies leading to delayed or foregone PPH benefits,^1^ and stresses the need for workforce training if the potential of PPH is to be realised.^1^ This position leaves us rather confused and raises still further questions. Pilot studies are usually the necessary antecedents of robust scientific studies of mechanisms, efficacy or effectiveness, and it is precisely in this realm that the evidence for PPH remains questionable or at least undercooked. In spite of this, PPH is front and centre in the UK Health Secretary’s forward strategy for personalised ‘predictive prevention’.^9^

What are the main scientific questions that should be answered before launching into workforce training and PPH implementation? Briefly, among the problems raised by PPH that we highlighted in a previous article are: (1) the inherent limits to risk prediction at the individual level, when the uncertainty due to random processes may be conflated with the uncertainty due to limited data or knowledge; (2) the constraints on prevention interventions that rely on individual agency, where such a focus might widen inequalities; (3) the undue emphasis on the supposed new insights gained from an individual’s genetics and their ‘big data’ instead of their own preferences for a particular intervention strategy (4) the diversion of scarce resources and attention away from the social determinants of health.^2^ Building on these issues, recent exchanges have called for a refocusing of the criticisms of PPH as scientific questions.^10^ In this essay, we, therefore, outline key scientific challenges relating to: defining risk for individuals; the limitations of current theory and study design for PPH and understanding the potential for unintended harms.

## Defining risk for individuals

We see significant problems in defining risks for individuals (Rose’s ‘causes of cases’) in areas where the epidemiology is just not up to the job. For example, large-scale ‘precision nutrition’ has been proposed as a PPH approach to delivering individualised dietary behavioural change in efforts to address obesity and the health impacts of poor diet.^11^ However, data to inform precision nutrition approaches are derived from population-level studies beset with problems of such magnitude that commentators have raised doubts about the possibility of ascertaining the impact of single dietary components on health outcomes.^12^ For example, if we usually have crushed walnuts with our low glycaemic index breakfast cereal and are subsequently observed to have a lower risk of heart disease, to what do we attribute our good fortune? Ioannidis is likely correct^12^ in contending that the implausible estimates of benefits or risks associated with specific dietary choices that we typically derive from population-level studies are due to the biases, residual confounding and selective reporting typical of these studies. While innovative approaches have been developed to address the key issue of collinearity among nutrients in our diet,^13^ these modern counterfactual based methods merely enable a more principled assessment of causal effects in populations, based on a clearer understanding of the assumptions coded in the underlying directed acyclic graph that the analyst proposes. Their relevance to us as individuals and our own risk, which may change as our dietary fastidiousness or budget changes over the life course, seems unclear.

## Limitations of current theory

Even if we were able to pinpoint an intervention target for an individual, how would we design and test the optimum intervention? What are the scientific questions and challenges that would need to be addressed in terms of theory and study design? The UK National Institute for Health and Care Excellence (NICE)^14^ and Public Health England^15^ both endorse the view that intervention design that is informed by sound theory has a better chance of success. Digital technologies that enable frequent, time intensive but tailored behavioural interventions hold some promise, but also pose many challenges to our current health behaviour theories and models.^16^

First, we lack a clear understanding and theory of factors influencing engagement with digital behavioural change interventions,^17^ which is a precondition for effectiveness for many of the interventions promoted through smartphones. Yardley *et al* rightly highlight the importance of considering how technological and behavioural elements combine to influence engagement.^17^ Extending our nutritional PPH example above, many time-varying factors (rushing to work, picking up the kids), not to mention personality traits (neuroticism or conscientiousness), may affect engagement with digital interventions to promote a healthy diet and health behaviours themselves. In addition, the behaviours may be subject to compensatory spillover effects affecting other behaviours at the level of the individual.^18^ Disentangling the influence of a single dietary component from these other influences on health outcomes in an individual is challenging, if not impossible.

Second, we will surely need to develop more complex mechanisms of behavioural change to explain within-person changes over time. By contrast, our current health behaviour theories have traditionally been formulated to explain between-person differences. Inherently, the processes affecting behavioural choices and actions within individuals over time are dynamic, and some have argued that lessons learnt from control systems engineering^16^ may help us accommodate feedback between underlying psychological constructs and so ‘close the loop’. Relatively scant attention has been given to developing a formal system of behavioural change theories, though the recent paper by West *et al* is an overdue and very welcome contribution in this regard.^19^ While commendable, the paper did not give much consideration to timescale and current theories appear to be a poor fit with the more rapid intraindividual dynamics of mobile technology interventions. Even if behavioural change theories can address timescale issues, current theory may be inadequate if, as some believe, behavioural change is better understood through the lens of chaos theory and complex adaptive systems,^20^ the key principles of which include unique sensitivity to initial conditions, leading to highly variable outcomes that are difficult to predict, occurring within adaptive systems where multiple components interact to produce results greater than the sum of their parts.

Ecological momentary assessment (EMA) has developed in response to the challenge of understanding behavioural change in individuals over time. EMA sets out to more intensively monitor behaviour and its antecedents over time by measuring moods and behaviours in real time, increasingly using ‘wearable’ technologies.^21^ The combination of EMA alongside smartphone behavioural change apps has contributed to the drive for digitally enabled personalised prevention and health promotion.^22^ But this is another area where, despite the policy hype, we believe that technological development may be outrunning the underpinning science.

Reporting standards for EMA studies have only recently been developed, and these should improve assessment of how measurement tools, sampling methods, sampling schedules, sample intensity, prompting strategies and compliance rates might affect study interpretation.^23^ The same authors developed a checklist and believe that when these key methodological considerations are reported accurately, we may begin to see improved reliability and validity of individual study findings.^23^ What this checklist does not offer is guidance on how best to *analyse* EMA data. A variety of statistical methods have been proposed to model and understand, or to predict, the behavioural dynamics in EMA settings, with different researchers opting for, variously, hidden multistate Markov models,^24^ time series modelling with Markov chains,^25^ dynamic regression and unified structural equation modelling.^26^ It would be too easy to dismiss such choices as matters of taste were it not for the fact that they usually materially affect the inferences that can be drawn.^27^

## Limitations of current study design

Assuming we have appropriated a theory informed optimal design for our PPH intervention, do we routinely use the right study designs to test its effectiveness? The conventional parallel group randomised controlled trial is not up to the task. The leading alternative designs, namely n-of-1 studies and microrandomised trials (MRTs) of Just in Time Adaptive Interventions (‘JITAI’) offer promise but raise ancillary scientific questions with which many researchers and practitioners have yet to grapple.^28^

N-of-1 studies provide the statistical basis to distinguish interindividual and intraindividual variation, which is crucial for intervention personalisation,^29^ offering a scientific basis for tailoring interventions to individuals. While the key design aspect of an n-of-1 study is that an individual is followed for a period of time and their responses measured before and after an intervention,^30^ there is in fact currently little consensus on the most effective way to analyse behavioural and psychological n-of-1 studies. In a recent systematic review of n-of-1 studies assessing behavioural change, only 25% of the 39 studies included in the review used appropriate statistical approaches for n-of-1 data analysis.^28 31 32^ The authors highlighted the challenges inherent in defining a null hypothesis for such studies or quantifying clinically important differences for power calculations.

Designing good tests of PPH interventions is dependent on defining the minimum clinically important difference (MCID) but population-level MICDs are different from individual MCIDs, both conceptually, and in the methods that must be leveraged for their estimation.^33^ This makes it challenging to define a ‘responder’^29^ in the context of personalised interventions. For example, we ourselves have shown how health professionals struggle to distinguish signal from noise when trying to identify ‘a responder’ to treatment in even simple scenarios.^34^ Some tend to overestimate the effects of a patients’ genotype on ‘responder’ status.^34^ But even if we properly define an individual-level MCID, the calculation of a final sample size (in this case how many epochs are necessary to observe and characterise the time trace of behaviour) will also depend on an individual’s engagement and willingness to be followed up and record data over prolonged periods. Less literate and less motivated subjects—more likely, perhaps, to be living in disadvantaged circumstances—are likely to have more missing data, and so the quality of predictions and estimates of intervention effectiveness may vary importantly across these groups.

Recently Murphy *et al* introduced the MRT design, which involves randomisation of interventions at various times and decision points at which an intervention may be effective, based on context or past behaviour.^35^ An example is the provision of a tailored activity suggestion to increase step count delivered via mobile phone technology.^36^ Microrandomisation of this JITAI for each participant on each day of the study permits an assessment of the causal effect on exercise of providing one intervention compared with an alternative, and whether this effect varies based on context or mood, which is being monitored (as per EMA) via the mobile phone.^35^ However, even Murphy *et al* have acknowledged that novel statistical methods are required to distinguish the effects of participant-determined features, dependent on personal agency, from those of routinely provided behavioural change intervention.^35^ This endogeneity problem is compounded when we acknowledge that awareness of being monitored could itself modify the experience and the behaviour—and how to fully address this in the analysis is not yet clear.^37^

Assessing the cost-effectiveness of PPH interventions poses an additional challenge. When new interventions are introduced to the UK National Health Service (NHS), NICE expects a robust cost effectiveness analysis, and adoption decisions are usually based on favourable incremental cost effectiveness ratios (ICERs). Ioannidis *et al* have pointed out some conceptual difficulties in applying population-level ICERs to individuals because for notionally the same ICER, cost-effectiveness will differ for individuals who have different priorities for specific outcomes, or who have different attitudes toward risk or time discounting.^38^ Methods are being developed to cater for such considerations,^39 40^ though it is not clear to us how the NHS should use this information in commissioning PPH. Developing a meta-analytical summary^41^ of a series of n-of-1 cost effectiveness studies would seem to defeat the purpose.

## Unintended consequences

A critical scientific (and practice) issue that needs greater deliberation must surely be the question of how to assess unintended consequences and harms in PPH interventions, and as part of their implementation—their ‘dark logic’.^42^ We can anticipate several unintended consequences that should be carefully monitored before widespread roll-out. In the bright new future of PPH, a key concern is that people may receive feedback about the supposed genetic or ‘environmental’ risk of multiple diseases. This feedback may have unpredictable effects. In the context of PPH based on genetic or digital phenotypes, excessive testing could lead to needless costs and potential harms, including false positive results, ‘overdiagnoses’ and unnecessary worry and intervention, a phenomenon that has been termed the ‘biomarkup’.^43^

Most of the genomic tests for risk prediction that have emerged from Genome Wide Association Studies for chronic disease lack long-term studies demonstrating robust evidence of improved outcomes or survival after testing. At the same time, the machine learning technology that is yielding new digital biomarkers from our ‘wearables’ is often based on algorithms that are not readily interpretable.^43^ One mildly ridiculous example is the commentary on recent mood prediction apps that offer such forecasts as ‘Happy with a 20% chance of sadness’, despite how little we know about how predicting mood could affect how people feel.^44^ But this problem may go beyond how people feel. A recent study showed that merely receiving genetic risk information (on obesity risk) changed individuals’ cardiorespiratory physiology and perceived satiety after food consumption^45^ and these effects were sometimes greater than the effects associated with actual genetic risk. Although this was a relatively small (though robust experimental) study, it is not unique. Others have shown how mindset can affect hormonal responses after food.^46^ Thus, when we convey genetic risk, we may in future have to take account of these physiological effects and adjust the threshold and nature of the intended intervention as appropriate. We suspect that the form of the risk communication, for example, relative versus absolute risk reduction, may itself have a bearing.

An equally serious point concerns the impact of artificial intelligence (AI)-based personalised interventions on health inequalities. AI-driven approaches to PPH lack an ‘effector arm’ that can grasp and handle the complexity of individuals’ lives.^47^ Machine learning algorithms let loose on ‘big data’ may simply amplify historical biases against vulnerable populations—it is contended that machine learning algorithms, model design, biases in data (based on engagement through personal agency) and the interactions of model predictions with patients or their doctors may prove important drivers of inequalities in future access to healthcare.^48^ And somewhat related to this is the risk that the unregulated realm of commercially sponsored apps for behavioural change that provide online or social media-enabled tailored ‘support’ may be susceptible to bots or targeted marketing from corporations whose products have little to offer personal or public health.^49^

## Conclusions

Before rushing headlong into implementation and workforce training for PPH, we need more insights into the problems and challenges outlined above. Until these issues are addressed, it will not be clear that the benefits of superficial intervention ‘tailoring’ for personalised prevention, common in today’s PPH interventions delivered through mobile technologies, outweigh the harms. Finally, in the current context of declining life expectancy and increasing inequalities in the UK,^50^ it is concerning that PPH approaches are being strongly promoted as solutions.^51^ We agree with the recent commentary by Olstad and McIntyre that PPH could benefit, conceptually and practically, from a refocus on the upstream social determinants of health.^52^ In the meantime, while evidence for sound policy and practice is still being sought, we consider Horton’s diagnosis of ‘pilotitis’ premature.^1^
